# Anatomical Assessment of the Thorax in the Neonatal Foal Using Computed Tomography Angiography, Sectional Anatomy, and Gross Dissections

**DOI:** 10.3390/ani10061045

**Published:** 2020-06-17

**Authors:** Alberto Arencibia, Juan Alberto Corbera, Gregorio Ramírez, María Luisa Díaz-Bertrana, Lidia Pitti, Manuel Morales, José Raduan Jaber

**Affiliations:** 1 Departamento de Morfología, Facultad de Veterinaria, Universidad de Las Palmas de Gran Canaria, Trasmontaña, Arucas, 35413 Las Palmas, Spain; joseraduan.jaber@ulpgc.es; 2 Instituto Universitario de Investigaciones Biomédicas y Sanitarias (IUIBS), Facultad de Veterinaria, Universidad de Las Palmas de Gran Canaria, Trasmontaña, Arucas, 35413 Las Palmas, Spain; juan.corbera@ulpgc.es (J.A.C.); manuel.morales@ulpgc.es (M.M.); 3 Departamento de Anatomía y Anatomía Patológica, Universidad de Murcia, 30100 Murcia, Spain; grzar@um.es; 4 Hospital Clínico Veterinario, Facultad de Veterinaria, Universidad de Las Palmas de Gran Canaria, Trasmontaña, Arucas, 35413 Las Palmas, Spain; luigi.bertrana@ulpgc.es (M.L.D.-B.); lpitti@fpct.ulpgc.es (L.P.)

**Keywords:** CT angiography, sections, dissections, thorax, anatomy, neonatal foal

## Abstract

**Simple Summary:**

This research aimed to describe the normal appearance of the thorax in neonatal foals by computed tomography angiography (CTA). The newborn foals were imaged using a 16-slice helical CT scanner after the administration of an iodinated contrast medium. CTA images and three-dimensional cardiac volume-rendered reconstructed images were obtained to enhance cardiovascular structures. In addition, thoracic anatomical sections and gross dissections were used as anatomical references. Clinically relevant anatomical structures were identified on the CTA images, anatomical sections, and gross dissections. These findings could serve as a reference to the CTA image assessment of the thorax of neonatal foals.

**Abstract:**

The purpose of this study was to correlate the anatomic features of the normal thorax of neonatal foals identified by CTA, with anatomical sections and gross dissections. Contrast-enhanced transverse CTA images were obtained in three neonatal foals using a helical CT scanner. All sections were imaged with a bone, mediastinal, and lung windows setting. Moreover, cardiac volume-rendered reconstructed images were obtained. After CT imaging, the cadaver foals were sectioned and dissected to facilitate the interpretation of the intrathoracic cardiovascular structures to the corresponding CTA images. Anatomic details of the thorax of neonatal foals were identified according to the characteristics of CT density of the different organic tissues and compared with the corresponding anatomical sections and gross dissections. The information obtained provided a valid anatomic pattern of the thorax of foals, and useful information for CTA studies of this region.

## 1. Introduction

CTA is a minimally invasive imaging technique used to assess the organs of the respiratory and cardiovascular system. In humans, medicine has become the imaging modality of choice for diagnosis of abnormalities, injuries, and thoracic disease [1,2]. CTA displays the anatomical detail of specific tissue densities and blood vessels more precisely compared with radiography, ultrasonography, and magnetic resonance imaging. It is due to its high spatial resolution, shortened examination time, and improved visibility of vascular structures and pulmonary parenchyma [3,4]. CTA can be used to obtain high-quality two-dimensional images and three-dimensional cardiac reformatted images that delineate the morphologic features of the cardiovascular system without the superimposition of other structures [5,6].

In equine medicine, radiology has been the main imaging modality used to image the thorax of foals, but the superimposition of adjacent anatomical structures makes its interpretation difficult [7]. Nonetheless, an advanced imaging technique such as the ultrasound [8,9] has given to the equine practitioners the opportunity to obtain an accurate diagnosis. In addition, helical CTA has also been used in horses, but information is limited to reports on the head, spinal cord, musculoskeletal system, and lungs [10,11,12,13,14,15,16,17,18,19]. To the best of our knowledge, a detailed anatomical study using CTA with an iodinated contrast medium for imaging the thorax of neonatal foals has not been reported before. 

An accurate anatomical interpretation of helical CTA of the thorax could be useful to aid in the diagnosis of different diseases described in neonatal foals such as thoracic trauma, pulmonary disorders, and congenital heart anomalies [20,21,22,23,24,25]. Therefore, the objectives of this study were: (1) To describe the normal cross-sectional anatomy of the thorax of neonatal foals using helical CTA images with the use of a vascular contrast medium, anatomic sections, and gross dissections, and (2) to obtain three-dimensional cardiac volume-rendered reconstructed images to assist in the understanding of the anatomy of the heart and the main intrathoracic vessels.

## 2. Materials and Methods 

### 2.1. Animals 

Three newborn crossbreed foals of 2, 5, and 6 days with weights ranging between 50–55 kg were selected from equine patients attending to the Veterinary Hospital of Las Palmas de Gran Canaria University between January to December 2019. The animals had neurological signs that included head tilt, seizures, circling, and ataxia. No other physical examination abnormalities were detected. After clinical evaluation, a combination of butorphanol 10 mg/mL at a dose of 0.4 mL (Torbugesic^®^; Zoetis S.L.U., Madrid, Spain), and dexmedetomidine 10 mg/mL at a dose of 0.3 mL (Dexdormitor^®^; Lab. Dr. Esteve SAU, Barcelona, Spain) injected IM were employed as a preanesthetic medication. Anesthesia using sevuflorane 98 (0.5 to 2%) (Sevoflo.; Abbot Laboratories SA, Madrid, Spain) was maintained during the procedure. After the head scan, the study of the thorax was performed. The euthanasia was done due to the diagnosis of CNS congenital abnormalities. The Animal Ethical Committee of Veterinary Medicine of Las Palmas de Gran Canaria University authorized the research protocol (MV–2016/04). The owners of these foals were informed of the study and signed a consent for participation in the study.

### 2.2. CTA Technique 

Contrast-enhanced sequential transverse CT slices were performed using a 16-slice helical CT scanner (Toshiba Astelion, Toshiba Medical System, Madrid, Spain). The animals were positioned symmetrically in dorsal recumbency on the CT couch and a standard clinical protocol (120 kVp, 80 mA, 512 × 512 acquisition matrix, 283 × 283 field of view, a spiral pitch factor of 0.94, and a gantry rotation of 1.5 s) was used to acquire transverse CT thorax images, with 3-mm slice thickness. In addition, all foals received a bolus of iomeprol 300 mg/mL at a dose of 2 mL/kg (Iomeron.; Rovi S.A., Madrid, Spain) via the jugular vein. The transverse original data was stored and transferred to the CT workstation. To better evaluate the CT appearance of the thoracic structures, three CT windows were applied by adjusting window widths (WW) and window levels (WL): A bone window setting (WW = 1500; WL = 300), a mediastinal window setting (WW = 248; WL = 123), and a lung window setting (WW = 1400; WL = −500). The original data were used to generate cardiac volume-rendered reconstructed images from the right and left surfaces, and the base of the heart after manual editing of the transverse CT images to remove bone structures and other soft tissues using a standard dicom 3D format (OsiriX MD, Geneva, Switzerland).

### 2.3. Anatomic Evaluation 

The interpretation of the CTA images was based on anatomical sections and gross dissections to facilitate the identification of the thoracic structures. At the end of the scanning procedure, the euthanized foals were frozen until solid. Later, two frozen cadavers were sectioned using an electric band saw to obtain sequential transverse anatomical sections. The other cadaver was used to perform the gross anatomical dissections within 24 h of death to minimize post-mortem changes. Thoracic structures studied in the CT images were correlated with those identified in the corresponding anatomical sections and gross dissections, evaluated according to the characteristics of CT density of different tissues and labelled to conform to anatomical texts [26,27].

## 3. Results

### 3.1. Transverse Computed Tomography Angiography Images

**Figure 1:**
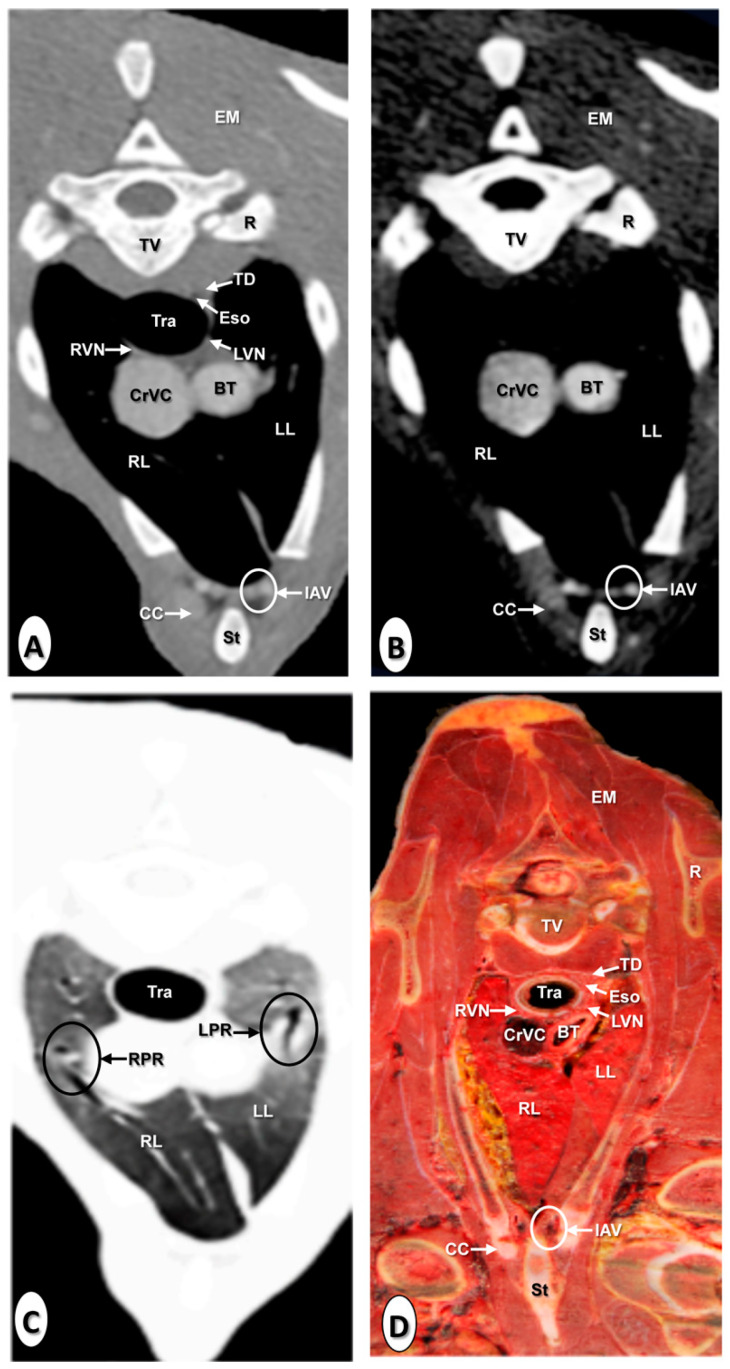
Transverse images at the level of the brachiocephalic trunk. (**A**) Computed tomography (CT) bone window; (**B**) CT mediastinal window; (**C**) CT lung window; and (**D**) anatomical section. These images are displayed so that the right side of the foal is to the viewer’s left and the dorsal view is at the top. Brachiocephalic trunk (BT); cranial vena cava (CrVC); epaxial muscles (EM); esophagus (Eso); internal thoracic artery and vein (IAV); left lung (LL); left pulmonary root (LPR); left vagus nerve (LVN); rib: Costal bone (R); rib: Costal cartilage (CC); right lung (RL); right pulmonary root (RPR); right vagus nerve (RVN); scapula (Sca); sternum (St); thoracic duct (TD); thoracic vertebra (TV); and trachea (Tra).

**Figure 2:**
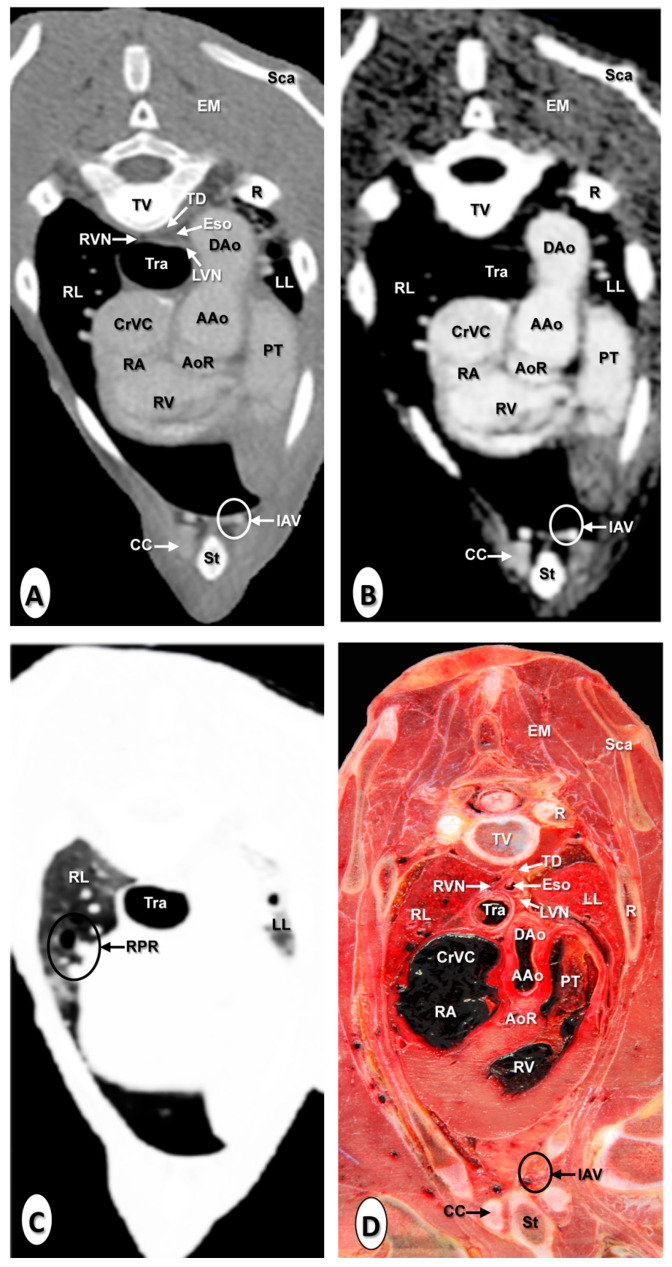
Transverse images at the level of the ascending aorta. (**A**) CT bone window; (**B**) CT mediastinal window; (**C**) CT lung window; and (**D**) anatomical section. These images are displayed so that the right side of the foal is to the viewer’s left and the dorsal view is at the top. Aortic root (AoR); ascending aorta (AAo); cranial vena cava (CrVC); descending aorta (Dao); epaxial muscles (EM); esophagus (Eso); internal thoracic artery and vein (IAV); left lung (LL); left vagus nerve (LVN); pulmonary trunk (PT); rib: Costal bone (R); rib: Costal cartilage (CC); right atrium (RA); right lung (RL); right pulmonary root (RPR); right vagus nerve (RVN); right ventricle (RV); scapula (Sca); sternum (St); thoracic duct (TD); thoracic vertebra (TV); and trachea (Tra).

**Figure 3:**
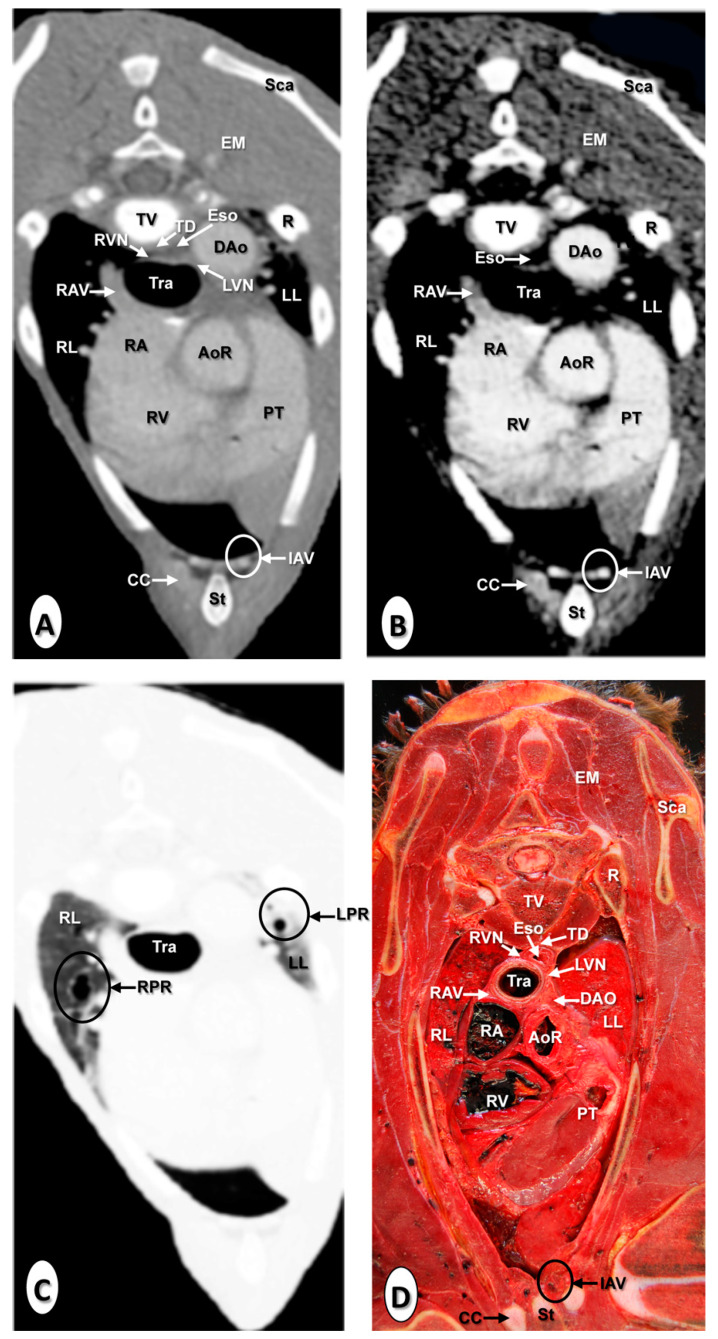
Transverse images at the level of the pulmonary trunk. (**A**) CT bone window; (**B**) CT mediastinal window; (**C**) CT lung window; and (**D**) anatomical section. These images are displayed so that the right side of the foal is to the viewer’s left and the dorsal view is at the top. Aortic root (AoR); descending aorta (Dao); epaxial muscles (EM); esophagus (Eso); internal thoracic artery and vein (IAV); left lung (LL); left pulmonary root (LPR); left vagus nerve (LVN); pulmonary trunk (PT); rib: Costal bone (R); rib: Costal cartilage (CC); right atrium (RA); right azygos vein (RAV); right lung (RL); right pulmonary root (RPR); right vagus nerve (RVN); right ventricle (RV); scapula (Sca); sternum (St); thoracic duct (TD); thoracic vertebra (TV); and trachea (Tra).

**Figure 4:**
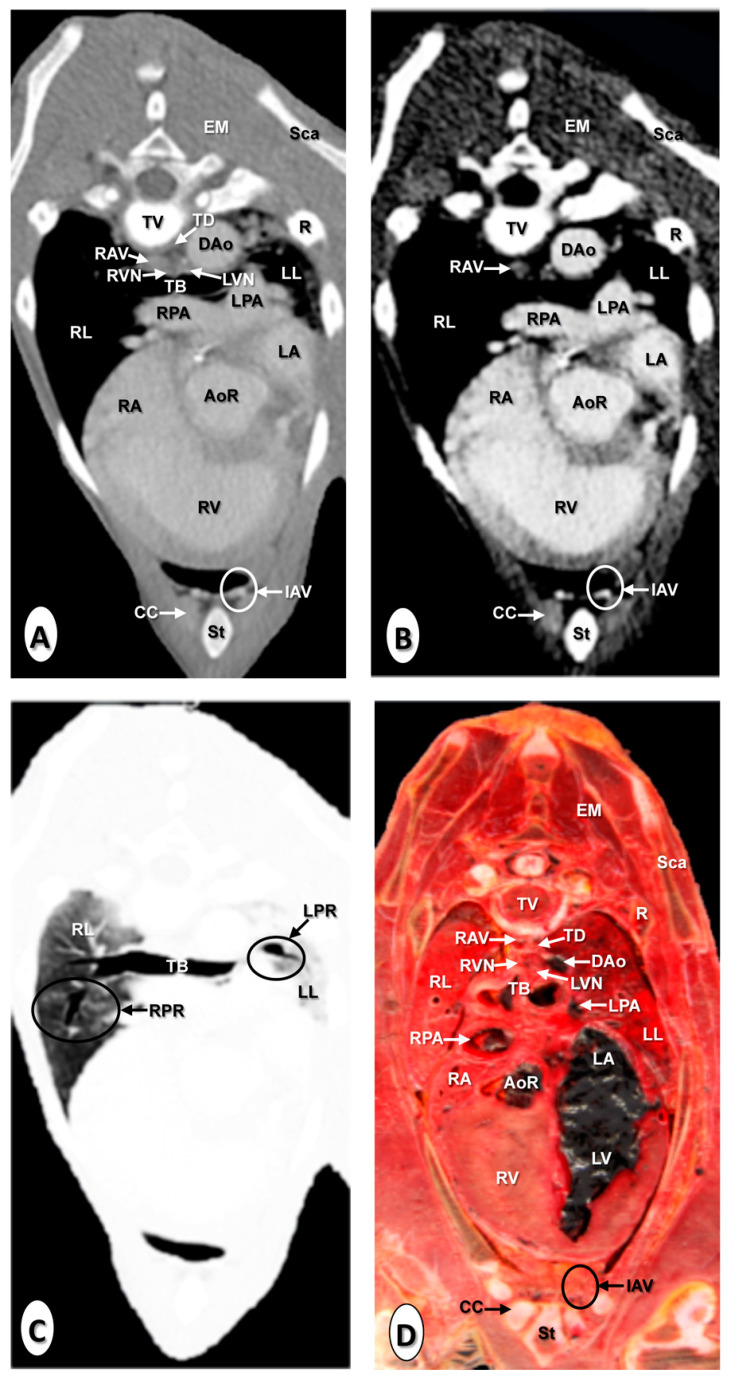
Transverse images at the level of the pulmonary arteries. (**A**) CT bone window; (**B**) CT mediastinal window; (**C**) CT lung window; and (**D**) anatomical section. These images are displayed so that the right side of the foal is to the viewer’s left and the dorsal view is at the top. Aortic root (AoR); descending aorta (Dao); epaxial muscles (EM); internal thoracic artery and vein (IAV); left lung (LL); left pulmonary artery (LPA); left pulmonary root (LPR); left vagus nerve (LVN); rib: Costal bone (R); rib: Costal cartilage (CC); right atrium (RA); right azygos vein (RAV); right lung (RL); right pulmonary artery (RPA); right pulmonary root (RPR); right vagus nerve (RVN); right ventricle (RV); scapula (Sca); sternum (St); thoracic duct (TD); thoracic vertebra (TV); and tracheal bifurcation (TB).

**Figure 5:**
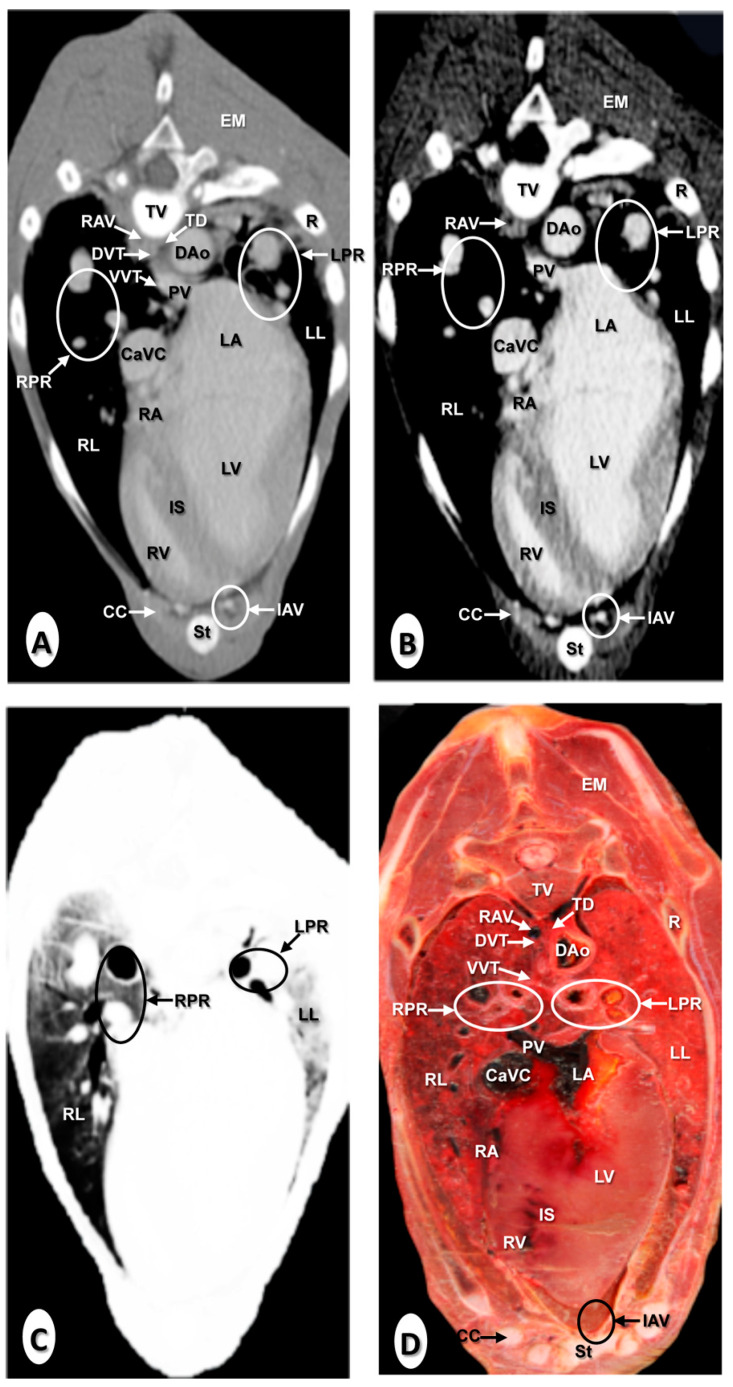
Transverse images at the level of the lung roots. (**A**) CT bone window; (**B**) CT mediastinal window; (**C**) CT lung window; and (**D**) anatomic section. These images are displayed so that the right side of the foal is to the viewer’s left and the dorsal view is at the top. Caudal cava vein (CaCV); descending aorta (Dao); dorsal vagal trunk (DVT); epaxial muscles (EM); internal thoracic artery and vein (IAV); left atrium (LA); left lung (LL); left pulmonary root (LPR); pulmonary vein (PV); rib: Costal bone (R); rib: Costal cartilage (CC); right atrium (RA); right azygos vein (RAV); right lung (RL); right pulmonary root (RPR); right ventricle (RV); scapula (Sca); sternum (St); thoracic duct (TD); thoracic vertebra (TV); and ventral vagal trunk (VVT).

**Figure 6:**
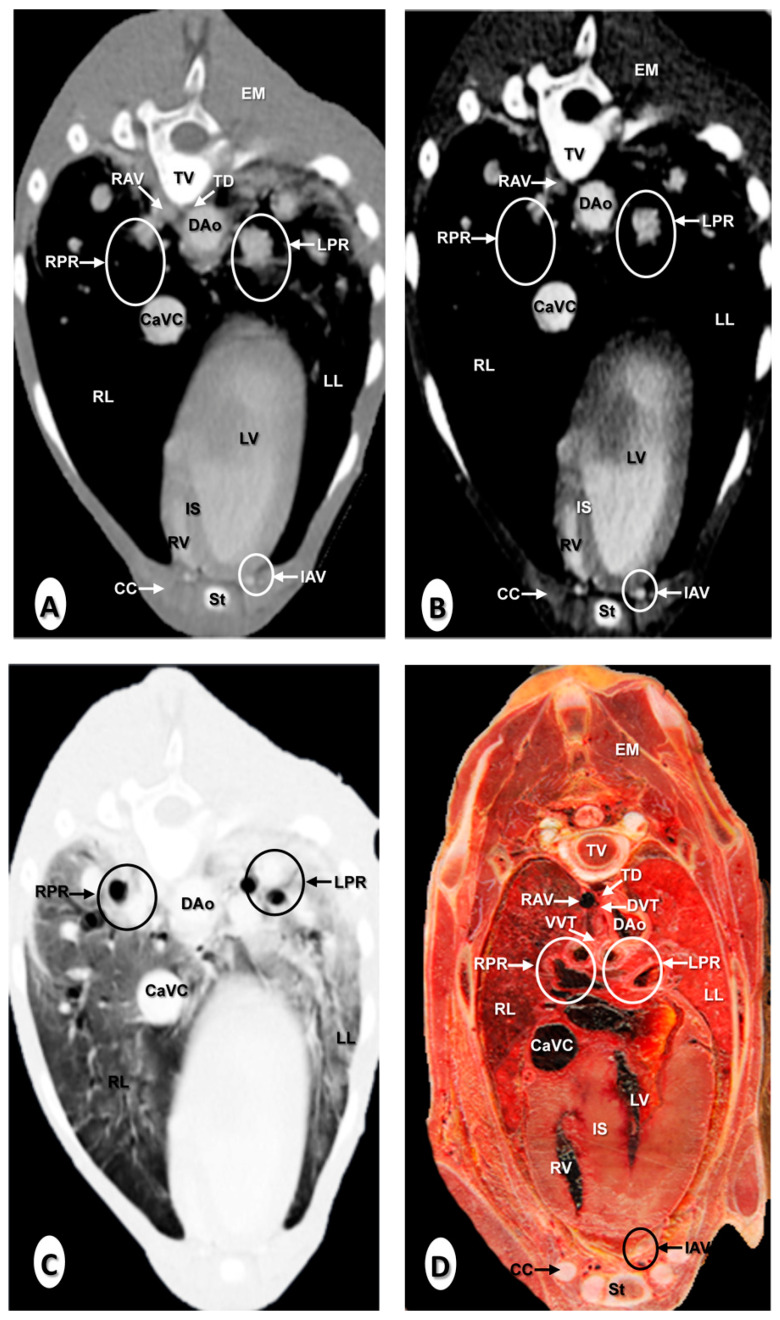
Transverse images at the level of the left ventricle. (**A**) CT bone window; (**B**) CT mediastinal window; (**C**) CT lung window; and (**D**) anatomical section. These images are displayed so that the right side of the foal is to the viewer’s left and the dorsal view is at the top. Caudal vena cava (CaVC); descending aorta (Dao); dorsal vagal trunk (DVT); epaxial muscles (EM); internal thoracic artery and vein (IAV); interventricular septum (IS); left lung (LL); left pulmonary root (LPR); left ventricle (LV). rib: Costal bone (R); rib: Costal cartilage (CC); right azygos vein (RAV); right lung (RL); right pulmonary root (RPR); right ventricle (RV); sternum (St); thoracic duct (TD); thoracic vertebra (TV); and ventral vagal trunk.

**Figure 7:**
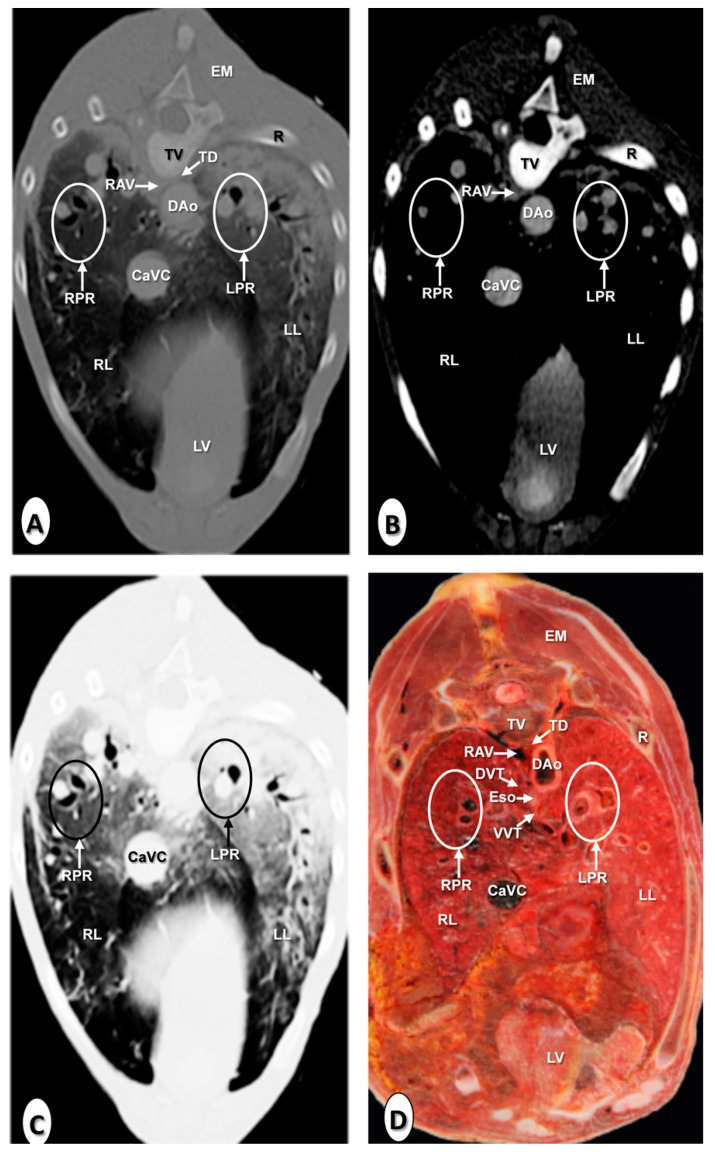
Transverse images at the level of the apex of the heart. (**A**) CT bone window; (**B**) CT mediastinal window; (**C**) CT lung window; and (**D**) anatomical section. These images are displayed so that the right side of the foal is to the viewer’s left and the dorsal view is at the top. Caudal vena cava (CaVC); descending aorta (Dao); dorsal vagal trunk (DVT); epaxial muscles (EM); left lung (LL); left pulmonary root (LPR); left ventricle (LV); rib: Costal bone (R); right azygos vein (RAV); right lung (RL); right pulmonary root (RPR); thoracic duct (TD); thoracic vertebra (TV); and ventral vagal trunk (VVT).

The results of the thoracic CTA images are presented in seven sequential transverse CTA images of the thorax at different levels that best correlated with the macroscopic sections (Figure 1, Figure 2, Figure 3, Figure 4, Figure 5, Figure 6 and Figure 7). Each figure consists of four images: (a) Bone window, (b) mediastinal window, (c) lung window, and (d) anatomical section. Transverse CT images are presented in a cranial to caudal progression from the level of the brachiocephalic trunk (Figure 1) to the level of the left ventricle and apex of the heart (Figure 7).

The CTA images obtained with the use of the bone window setting (Figure 1A, Figure 2A, Figure 3A, Figure 4A, Figure 5A, Figure 6A and Figure 7A), provided a good differentiation between the bones and the soft tissues of the thoracic cavity. Thus, the thoracic vertebrae (including the vertebral body and arch, and the corresponding articular, transverse, and spinous processes), the ribs (with its head tubercle, body, and costal cartilage), and the sternum were well visualized (Figure 1A, Figure 2A, Figure 3A, Figure 4A, Figure 5A, Figure 6A and Figure 7A). In addition, the vertebral cortical and bone marrow fat were also delineated. However, the costovertebral, costochondral, and sternocostal joints and those muscles associated with the thorax such as epaxial (semispinalis, longissimus, and iliocostalis) and thoracic wall (external and internal intercostal, and pectoral) muscles appeared with an intermediate CT density (Figure 1A, Figure 2A, Figure 3A, Figure 4A, Figure 5A, Figure 6A and Figure 7A). Other anatomical structures such as the thoracic duct (Figure 1A, Figure 2A, Figure 3A, Figure 4A, Figure 5A, Figure 6A and Figure 7A), the right and left vagus nerves (Figure 1A, Figure 2A, Figure 3A and Figure 4A), and the dorsal and ventral vagal trunks (Figure 5A) were also identified.

The CT mediastinal window (Figure 1B, Figure 2B, Figure 3B, Figure 4B, Figure 5B, Figure 6B and Figure 7B) showed good visualization of the bony thoracic wall structures. Moreover, this CT window also provided an excellent visualization of the heart with its chambers (atrium and ventricles) and the main arteries and veins, which appeared with a high attenuation due to the intravenous contrast medium (Figure 1B, Figure 2B, Figure 3B, Figure 4B, Figure 5B, Figure 6B and Figure 7B). Thus, important associated vessels such as the cranial (Figure 1B and Figure 2B), and the caudal vena cava (Figure 5B, Figure 6B and Figure 7B) were seen leading into the right atrium. Additionally, the course of the right azygos vein (Figure 3B Figure 4B, Figure 5B, Figure 6B and Figure 7B), the pulmonary trunk (Figure 3B), and the left and right pulmonary arteries (Figure 4B) were observed. Other intrathoracic vessels, including the aortic root (Figure 2B, Figure 3B and Figure 4B), ascending (Figure 2B) and descending aorta (Figure 2B, Figure 3B, Figure 4B, Figure 5B, Figure 6B and Figure 7B), and the internal thoracic arteries and veins (Figure 1B, Figure 2B, Figure 3B, Figure 4B, Figure 5B and Figure 6B) were also identified. By contrast, with the use of CT bone window images (Figure 1A, Figure 2A, Figure 3A, Figure 4A, Figure 5A, Figure 6A and Figure 7A), these structures appeared with an intermediate CT density. Other structures such as the myocardial walls showed an intermediate CT density and were best visualized in the CT bone and mediastinal windows settings (Figure 1A, Figure 2A, Figure 3A, Figure 4A, Figure 5A, Figure 6A, Figure 7A and Figure 1B, Figure 2B, Figure 3B, Figure 4B, Figure 5B, Figure 6B, Figure 7B).

Concerning the lungs, the CT bone (Figure 1A, Figure 2A, Figure 3A, Figure 4A, Figure 5A, Figure 6A and Figure 7A) and mediastinal (Figure 1B, Figure 2B, Figure 3B, Figure 4B, Figure 5B, Figure 6B and Figure 7B) window settings showed the bronchi and the vascular formations of the lungs, which were only clearly defined at the level of the hilus because of the deeper lumen and the use of intravenous contrast medium. In contrast, the CT lung window (Figure 1C, Figure 2C, Figure 3C, Figure 4C, Figure 5C, Figure 6C and Figure 7C) allowed a better definition of the lobes and a better tomographical definition of the trachea, tracheal bifurcation, main bronchi, and lobar bronchi due to these structures that presented higher attenuation than the lungs. Moreover, it was possible to visualize the triad that comprises the lobar pulmonary vein, the lobar arterial branch, the lobar bronchus, and the pleural cavity. 

### 3.2. Anatomical Sections

On transverse anatomical sections (Figure 1D, Figure 2D, Figure 3D, Figure 4D, Figure 5D, Figure 6D and Figure 7D), additional morphologic and topographic information about the thoracic structures could be identified when compared with CTA images. All bones, cartilaginous structures, and associated muscles were identified. The respiratory tract structures, including the trachea (Figure 1D, Figure 2D and Figure 3D) and its bifurcation (Figure 4D), the principal and lobar bronchi, and pulmonary parenchyma (Figure 5D, Figure 6D and Figure 7D), were also well observed. Other intrathoracic structures such as the heart with its chambers and associated large vessels were likewise visible in Figure 1D, Figure 2D, Figure 3D, Figure 4D, Figure 5D, Figure 6D and Figure 7D. Other anatomical structures such as the thoracic duct (Figure 1D, Figure 2D, Figure 3D, Figure 4D, Figure 5D, Figure 6D and Figure 7D), the right and left vagus nerves (Figure 1D, Figure 2D, Figure 3D and Figure 4D), and the dorsal and ventral vagal trunks (Figure 5D, Figure 6D and Figure 7D) were identified.

### 3.3. Gross Anatomical Dissections

**Figure 8:**
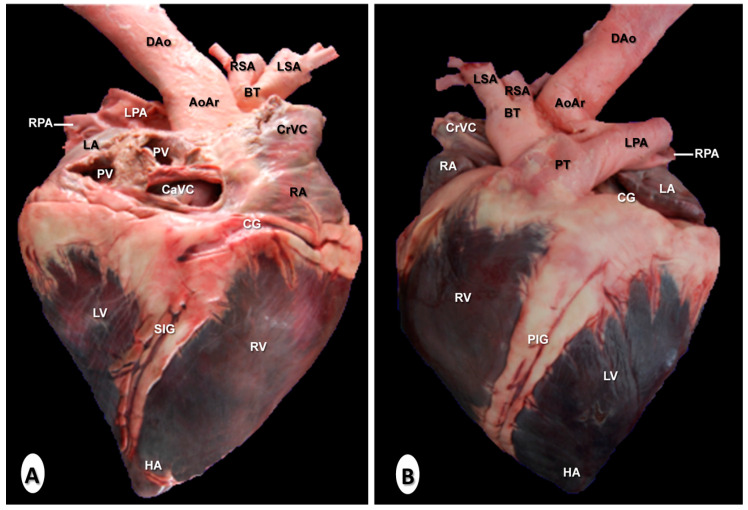
Anatomical dissections of the heart. (**A**) Atrial surface and (**B**) auricular surface. Aortic arch (AoAr); brachiocephalic trunk (BT); caudal vena cava (CaVC); cranial vena cava (CrVC); descending aorta (DA); heart apex (HA); left atrium (LA); left pulmonary artery (LA); left subclavian artery (LSA); left ventricle (LV); paracoronary interventricular groove (PIG); pulmonary trunk (PT); pulmonary vein (PV); right atrium (RA); right pulmonary artery (RPA); right subclavian artery (RSA); subsinuosal interventricular groove (SIG); and right ventricle (RV).

Figure 8 is a composition of two anatomical gross dissections at the level of the atrial (Figure 8A) and auricular (Figure 8B) surfaces of the heart. All chambers, grooves, and the main blood vessels were identified. Thus, the myocardial walls and the coronary groove were well visualised in both images (Figure 8A,B), as well as the subsinuosal interventricular (Figure 8A) and the paracoronary interventricular (Figure 8B) grooves. The location relative to the cranial and caudal vena cava leading into the right atrium could be clearly observed in all views of the heart (Figure 8A,B). In addition, the pulmonary veins were identified in the image corresponding to the atrial surface of the heart (Figure 8A), while the pulmonary trunk arising from the right ventricle was clearly visible in Figure 8B. In addition, the right and left pulmonary arteries were also well identified (Figure 8). The course of the ascending aorta arising from the left ventricle, and the main branches such as the brachiocephalic trunk, the right, and left subclavian arteries, and the descending aorta were also easily identified (Figure 8B). 

### 3.4. Cardiac Volume-Rendered Reconstructed CTA Images

**Figure 9:**
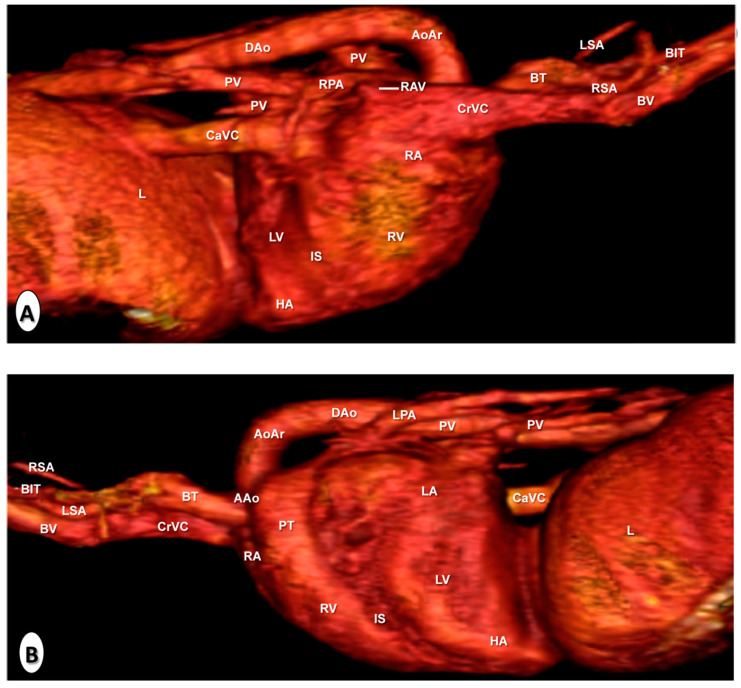
Three-dimensional volume-rendered reconstructed images of the normal neonatal foal heart and associated blood vessels. Parasagittal aspects. (**A**) Right lateral aspect and (**B**) left lateral aspect. Aortic arch (AoAr); ascending aorta (AAo); bicarotid trunk (BIT); brachiocephalic trunk (BT); brachiocephalic vein (BV); caudal vena cava (CaVC); cranial vena cava (CrVC); descending aorta (Dao); heart apex (HA); left atrium (LA); left pulmonary artery (LPA); left subclavian artery (LSA); left ventricle (LV); liver (L); pulmonary trunk (PT); pulmonary vein (PV); right atrium (RA); right azygos vein (RAV); right pulmonary artery (RPA); right subclavian artery (RSA); and right ventricle (RV).

**Figure 10:**
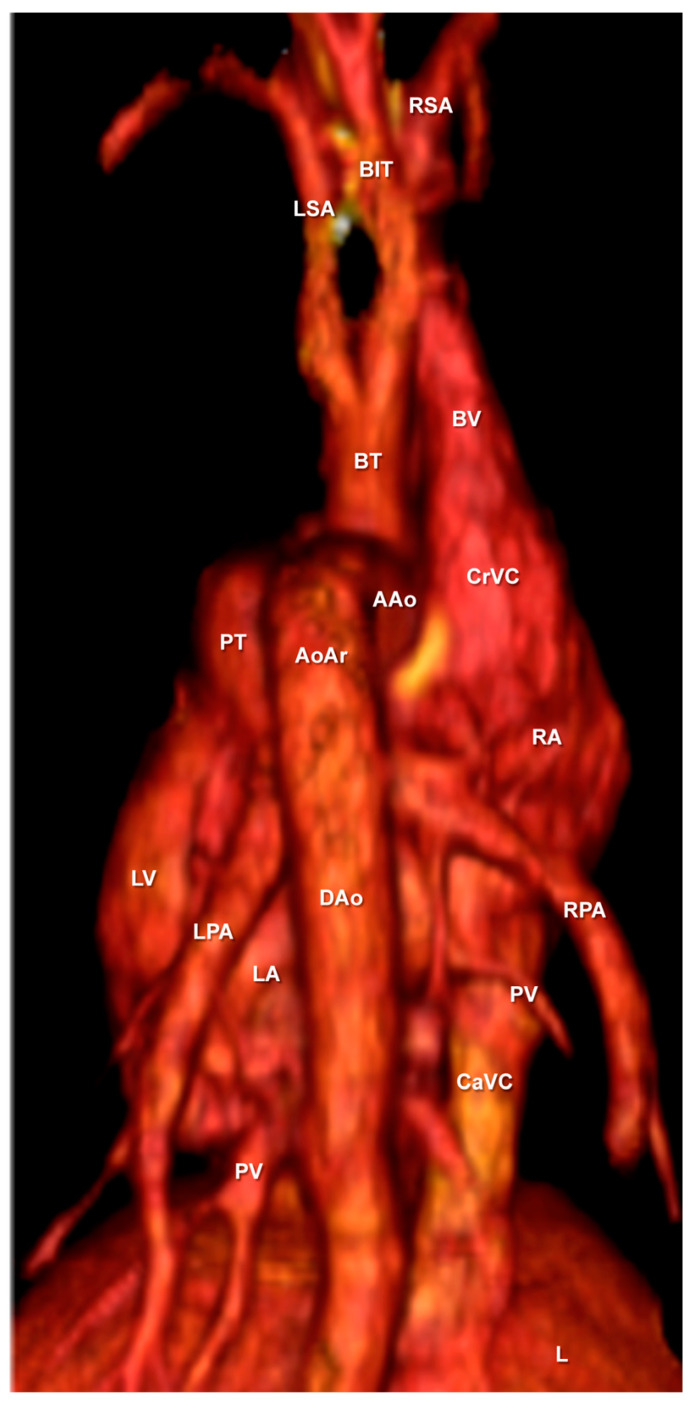
Three-dimensional volume-rendered reconstructed image of the normal neonatal foal heart and associated blood vessels. Dorsal aspect. Aortic arch (AoAr); ascending aorta (AAo); bicarotid trunk (BIT); brachiocephalic trunk (BT); brachiocephalic vein (BV); caudal vena cava (CaVC); cranial vena cava (CrVC); descending aorta (Dao); left atrium (LA); left pulmonary artery (LPA); left subclavian artery (LSA); left ventricle (LV); liver (L); pulmonary trunk (PT); pulmonary vein (PV); right atrium (RA); right azygos vein (RAV); right pulmonary artery (RPA); and right subclavian artery (RSA).

Cardiac three-dimensional volume-rendered reconstructed images corresponding to right (Figure 9A) and left lateral surfaces (Figure 9B) and the base (Figure 10) of the heart are presented. Volume-rendered reconstructed CTA images provided a good visualisation of the heart and the major associated vessels. Thus, the cardiac chambers and the main associated blood vessels were identified in all CT reconstructed images (Figure 9 and Figure 10). The location relative to the cranial and caudal vena cava leading into the right atrium could be clearly observed on all views of the heart (Figure 9 and Figure 10). Other important vessels such as the right azygos and brachiocephalic veins joined to the cranial vena cava were seen (Figure 9 and Figure 10). In addition to these observations, the junction of the pulmonary veins entering into the left atrium was identified in all volume reconstructed CT images (Figure 9 and Figure 10). Figure 9 (panel A) shows the course of the pulmonary trunk originating from the right ventricle. In contrast, the pulmonary artery bifurcation (right and left pulmonary arteries) was clearly identified on the dorsal aspect (Figure 10). The course of the ascending aorta arising from the left ventricle (Figure 9B and Figure 10), and its main branches (such as the descending aorta and brachiocephalic trunk) were easily identified in Figure 9B and Figure 10. The cranial branches of the brachiocephalic trunk such as the left and right subclavian arteries, and bicarotid trunk could be identified on all reconstructed CT images (Figure 9 and Figure 10).

## 4. Discussion

In humans, advanced image-based diagnostic techniques, especially helical computed tomography angiography makes possible the evaluation of the cardiac and vascular thoracic structures due to its fast imaging acquisition, the acquisition of body sections from different tomographic planes, good anatomic resolution without superimposition, high contrast between different vascular structures, and excellent tissue-like differentiation [1,2,3,4]. In addition, the use of CTA allows the obtention of three-dimensional volume-rendered reconstructed images that provide excellent detail of the heart, and the arteries and veins of this region [1,5,6].

In veterinary medicine, the use of third or four generations of CT scanners has provided an excellent anatomic resolution of the thoracic structures [28,29]. In the present study, CTA images were obtained using a helical CT scanner that provided a qualitative overview of thoracic morphology, giving adequate information of midline thoracic vascular structures, a good depiction of the four chambers of the heart, as well as serving of a standard reference for the size and positions of the heart and main blood vessels. The use of a 16-slice configuration CT scanner and a similar protocol was reported in other studies performed in humans [1,2,3,4,5,6], neonatal foal [17,18,19], dog [30,31], cat [32,33], and goat [34]. 

Clinical evaluation of the equine thorax is laborious due to its anatomical complexity, which makes it difficult to diagnose diseases by physical examination and conventional diagnostic techniques. Nevertheless, advanced diagnostic techniques as CTA has shown considerable advantages over traditional imaging techniques since it gives an accurate anatomical detail of blood vessels, higher differentiation of tissue densities [30,31]. In addition, CTA is more sensitive in detecting diseases such as congenital abnormalities of the cardiovascular system including the heart, vascular malformations, injuries, tumors, aneurysms, vessels ruptures or tears, and pulmonary embolism [1,2,3,4,5,32].

In this research, an intravascular contrast medium administration was very helpful to identify the heart chambers, the main associated vessels, and the delineation of the adjacent non-vascular structures. In veterinary medicine, only a few studies have applied contrast-enhanced helical CT to perform anatomical or clinical studies of the thoracic cavity in dogs [31,35,36,37] and cats [32], as well as in other studies performed in the thorax [17,18,19] and abdomen [38] of foals. Nevertheless, to our knowledge, the use of intravenous contrast agents to describe the normal anatomy of the thorax in neonatal foals has not been reported before. In CT imaging, the use of an appropriate window width is a key to successful diagnosis [30,31]. In the present study, thoracic CTA images were evaluated by the use of bone, mediastinal, and lung window settings. The CT bone window provided some valuable anatomical information of the cortical and medullar marrow fat of the bones, whereas the CT mediastinal window provided an excellent detail of soft tissues, especially the heart and the major associated blood vessels. By contrast, the lung window setting gave a better definition of the respiratory tract and intrapulmonary vascular structures.

The images obtained by volume-rendered tomographic reconstruction are the most flexible 3D visualization tools [1,35,36,37]. In our study, the contrast CT volume-reconstructed images performed by the post-processing bone removal technique provided an excellent anatomical detail of the lateral and dorsal aspects of the heart and main associated vessels. Lateral CT reconstructed acquisitions were preferred for the evaluation of the anatomic relationships between the heart chambers and the main blood vessels, while the dorsal view was selected for identification of midline thoracic vascular structures because it yielded detailed information about the pulmonary vessels, and the main branches of the brachiocephalic trunk. Usually, motion artifacts make it difficult to identify various parts of the heart or the lungs on CT images [28]. In this study, the use of helical scanning equipment tomography on living foals minimized the artifacts generated by cardiovascular and respiratory movements. However, its use in equine medicine is currently limited because of its expense, availability, and complications of acquiring CT images in older foals and adult horses due to their physical size [17,18,19]. 

This CTA anatomic study has confirmed the valid use of cadavers to evaluate different anatomic patterns. The absence of blood flow in dead animals must be taken into account when compared with live specimens. Results from the current study showed that the use of frozen anatomical sections was helpful in the identification of different thoracic structures observed on transverse CTA images and guaranteed the matching accuracy. In addition, the identification of vascular structures of the foal thorax in the volume-rendered reconstructed CTA images were facilitated by gross anatomical dissections of the atrial and auricular surfaces of the heart. Therefore, the three foals used in this study showed cardiovascular anatomy similar to that described in the anatomical literature [26,27]. Thus, the main anatomical differences in the cardiovascular structure of equines compared to dogs such as the subclavian arteries and bicarotid trunk arising from the brachiocephalic trunk could be distinghished. 

There are no previously published anatomic identifications as these reported in this study, which could be applied as an initial anatomic approximation to other CTA studies on foals. Therefore, the information provided could be used for the evaluation of CT images of foals with thoracic disease. In humans, new CT scanners have achieved improved diagnostic capabilities to evaluate a wide variety of congenital and acquired heart diseases [1,2,3,4,5,6]. With improvements in CT protocols and optimized scanners, CT angiography images will become an accurate method for evaluating the foal thorax [17,18,19], and in the diagnosis of several thoracic diseases described in equine medicine [20,21,22,23,24,25].

## 5. Conclusions

Helical CT images provided adequate detail of the thorax of normal neonatal foals and were a useful imaging modality for anatomical evaluation. This information could serve as an initial anatomic reference aid to clinicians for the diagnosis of suspected thorax-associated diseases in foals.
